# Enhancing allocation of visual attention with emotional cues presented in two sensory modalities

**DOI:** 10.1186/s12993-022-00195-3

**Published:** 2022-09-22

**Authors:** Ulrike Zimmer, Mike Wendt, Marlene Pacharra

**Affiliations:** 1 Faculty of Human Sciences, Department of Psychology, MSH Medical School Hamburg, Hamburg, Germany; 2 ICAN Insitute of Cognitive and Affective Neuroscience, MSH Medical School Hamburg, Hamburg, Germany; 3 Faculty of Psychology, Department of Biopsychology, Institute of Cognitive Neuroscience, Ruhr-University Bochum, Bochum, Germany

**Keywords:** Spatial attention, Multisensory, Emotion, Sensory modality, Fear, EEG, ERP

## Abstract

**Background:**

Responses to a visual target stimulus in an exogenous spatial cueing paradigm are usually faster if cue and target occur in the same rather than in different locations (i.e., valid vs. invalid), although perceptual conditions for cue and target processing are otherwise equivalent. This cueing validity effect can be increased by adding emotional (task-unrelated) content to the cue. In contrast, adding a secondary non-emotional sensory modality to the cue (bimodal), has not consistently yielded increased cueing effects in previous studies. Here, we examined the interplay of bimodally presented cue content (i.e., emotional vs. neutral), by using combined visual-auditory cues. Specifically, the current ERP-study investigated whether bimodal presentation of fear-related content amplifies deployment of spatial attention to the cued location.

**Results:**

A behavioral cueing validity effect occurred selectively in trials in which both aspects of the cue (i.e., face and voice) were related to fear. Likewise, the posterior contra-ipsilateral P1-activity in valid trials was significantly larger when both cues were fear-related than in all other cue conditions. Although the P3a component appeared uniformly increased in invalidly cued trials, regardless of cue content, a positive LPC deflection, starting about 450 ms after target onset, was, again, maximal for the validity contrast in trials associated with bimodal presentation of fear-related cues.

**Conclusions:**

Simultaneous presentation of fear-related stimulus information in the visual and auditory modality appears to increase sustained visual attention (impairing disengagement of attention from the cued location) and to affect relatively late stages of target processing.

## Background

In daily life, emotional stimuli, such as a fear-evoking dog, often feature salient properties in more than one sensory modality (e.g., sound of barking and sight of aggressive posture). What are the consequences of such bimodally corresponding signals for the deployment of attention to the stimulus object they belong to?

The most relevant experimental protocol for investigating stimulus-elicited allocation of attention is referred to as the spatial cueing paradigm. In this protocol, a target stimulus is presented either at a location indicated by a preceding cue (i.e., valid condition) or at a different location (i.e., invalid condition) which can be considered equivalent concerning other factors of perceptual relevance. In studies of exogenous spatial cueing, the cue is presented at one of the potential target locations (usually one of them located on the left side and one located on the right side of a central fixation point). Studies of exogenous spatial cueing reliably yielded faster responses in valid than in invalid conditions [32, 54]. This cueing validity effect has been ascribed to the deployment of attention to the cued location. Such findings are not confined to situations, in which cue and target belong to the same sensory modality but have also been observed in studies, in which an unisensory cue and an unisensory target consisted of stimuli presented in two different modalities (e.g., an auditory cue with a visual target), demonstrating crossmodal cueing of spatial attention [5, 10, 67]. In contrast to crossmodal presentations, studies of bimodal spatial cueing have been conducted to investigate the combined effects of attentional cues presented simultanesouly in two sensory modalities (e.g., a visual-auditory cue).

Although some bimodal spatial cueing studies (audio-visual cues preceding visual targets) have been conducted (e.g., [47, 63]), these studies did not investigate shifts of spatial attention evoked by emotion. In an ERP study [63], laterally presented bursts of white noise and LED flashes served as cues that could be presented alone or together (on the same side). These unisensory or bimodal cues preceded triangular shaped LED-flashes presented at the corresponding (valid) or opposite (invalid) side, which had to be categorized as pointing up or down. There were no gains for bimodal versus unimodal cueing presentations on target-processing neither behaviorally nor in terms of any target-related ERP-effect. However, ERP-results indicated a clearly enhanced amplitude of the cue-elicited P1 over contralateral visual areas related to the processing of bi- versus unimodal cues. According to the authors, the discrepancy of target-related behavioral and cue-related ERP results could be explained by the fact that target discrimination follwing the bimodal cue was not demanding enough to require specific shifts of spatial attention. Another study involving bimodal spatial cueing used bilateral presentation of visual cues (i.e., the face of a cat located on one side, and the face of a dog located on the other side of the screen) and added an animal sound, presented via a centrally located loudspeaker [47]. Follwing such a bimodal cue, a visual target (Gabor-patch) was unpredictively presented to 50% on the side indicated by the cueing-picture matching the cueing sound. The task consisted of easy and difficult orientation changes in the visual Gabor-patch targets. The authors found that responses to targets were faster to the side where the animal sound matched with the animal picture, but this was confined to trials featuring high demands of target discrimination. This result prompted the authors to suggest that bimodal enhancement of spatial cueing depends on the amount of perceptual load.

On the other hand, several spatial cueing studies investigated emotional shifts of spatial attention by using unisensory or crossmodal (i.e., cue and target of different sensory input) fear-related or anger-related cues (i.e., faces or voices). These studies demonstrated larger validity differences on the behavioral level with emotionally compared to neutral cues [7, 23]. Extant findings also suggest that the direction of an attentional shift by an emotional cue depends on its specific emotional content. For example, while fearful facial expressions appear to attract attention toward their location, disgusted expressions were associated with shifts of attention away from their location, resulting in a reversed validity effect (i.e., faster responding in invalid than in valid trials [44, 72]). Noteably, a similar pattern was found when using sound cues of fearful and disgusting content [74]. These results point to a modality independence of fear-related attraction of attention and disgust avoidance, suggesting that the modulation of attentional deployment is evoked by the semantic content rather than by superficial perceptual features of an emotional stimulus.

Corresponding ERP data for emotional spatial cueing demonstrated that the fear-related attraction of attention can result in larger validity differences of P1-activation for targets presented after fearful or angry compared to neutral facial or voice cues [8, 55, 56]. Further, the target-related P3-component, a positive ERP peaking around 300–400 ms after target onset over occipital-parietal electrodes, has been observed to be inversely correlated with the behavioral validity effects associated with the two types of emotional cue content [21, 56, 73–75]. This inverse P3-modulation corresponds to a redirection of spatial attention to the target, when a target followed a cue on the previously unattended side (cf. [46]). In summary, whereas spatial cueing effects tend to be enhanced by emotional cue content, redundant bimodal cueing yielded a more complex pattern indicating ERP-effects such as a modulation of the P1-component which were not always accompanied by corresponding behavioral effects (possibly depending on task demands as assumed by [63]).

Spatial cueing with peripherally presented cues is sensitive to the timing of stimulus presentation. Specifically, the validity effect tends to decrease and often reverse (i.e., faster responses in invalid compared to valid conditions) when the interval separating cue and target is extended, a phenomenon referred to as *Inhibiton of Return* (*IOR*, [54], see [37], for an overview). IOR is of particular relevance for studies investigating cueing effects of auditory features which cannot be identified instantly. In contrast to a remarkably fast emotional recognition of visual facial expressions (17–100 ms; [72]) or identification of elementary auditory features, emotional voice recognition requires temporal integration of stimulus properties changing dynamically across an extended period of time [64]. In practice, the duration of an auditory fear-related voice needs to be considerably longer than 600 ms to ensure a high level of correct identification if the task requires distinguishing it from other emotional or neutral voices [22, 53].

Despite of using a long stimulus duration of 1000 ms for voice cues in a previous study [75], we observed typical cueing validity effects when visual targets followed neutral voice cues after a short interstimulus-interval (ISI, i.e., time between cue offset and target onset). In contrast, validity effects after neutral voices reversed following a long ISI, possibly indicating IOR. These findings suggest that (at least for auditory cues) a long ISI rather than a long cue-target onset asynchrony may be crucial for IOR to occur. Taken together, adding identifiable emotional content to an auditory cue requires a sufficiently long voice duration to enable content identification, which, however, does not itself evoke IOR-effects as long as combined with short ISI (cf. [75]).

In spatial cueing paradigms, P1-activity as well as N170-activity, time-locked to visually presented face cues, has often been increased when comparing emotional facial expressions to neutral ones (P1: [15], N170: e.g., [11, 69], see [31] for a review). Importantly, the P1 increase associated with emotional cue content seemed to be independent of the type of stimulus used as cues as a similar P1-enhancement was also found when comparing frightening versus neutral pictures of objects other than faces (i.e., IAPS—pictures, [9, 38]). The N170 component is a component typically observed in connection with processing face (-like) stimuli which originates from the fusiform face area (FFA) [26]. The increase of N170 activity evoked by emotional content occurred independently of specific task demands (i.e., passive viewing vs. detection/discrimination) ([25, 60, 66, 71], see [31] for a review). So far, there seems to be no evidence for an additional enhancement of the N170 evoked by emotional faces when accompanied by vocal utterances matching the displayed emotion. Conversely, angry vs. neutral vocal expressions, presented during face encoding, reduced P1 and N170 to matching facial expressions [43].

**Fig. 1:**
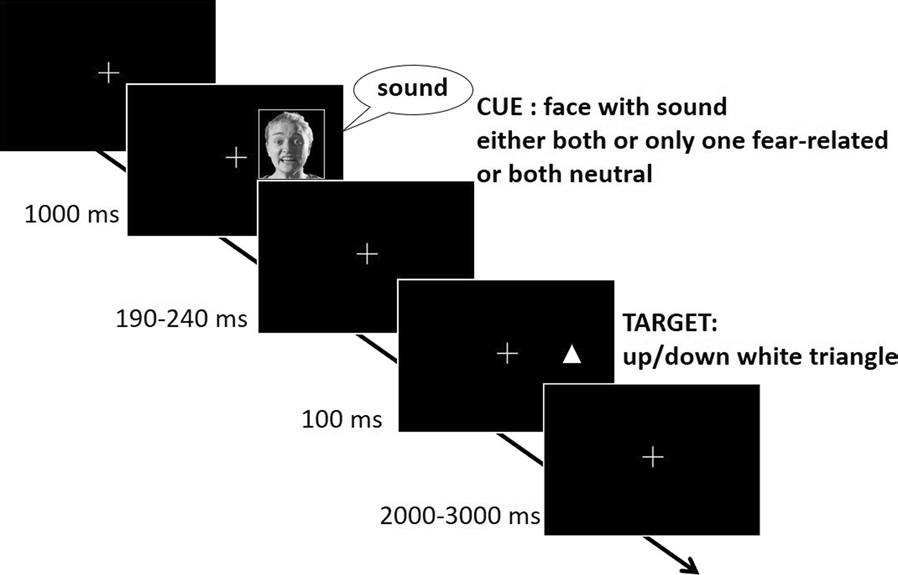
Task paradigm. A schematic example of a valid stimulus sequence is shown. Bimodal face-voice cues consisted of either both fearful, one fearful (paired with neutral) or both neutral contents. 50% of the targets followed each cue type on the same side (valid) and 50% on the opposite side (invalid). Participants were required to push one of two buttons indicating the pointing direction (up/down) of the target triangle

In the present study, we used a spatial cueing paradigm with bimodal face-voice cues that varied concerning their degree of emotional content; i.e., fearful in face and voice (double emotional cue), fearful voice/neutral face or fearful face/neutral voice (single emotional cue), or both neutral (neutral cue). The aim was to investigate whether double emotional face-voice cues attract attention to their location more strongly than single emotional cues or purely neutral cues, respectively. If true, double emotional cues should elicit an enhanced validity effect in behavior (reaction times, hit rates) as well as more pronounced target-related ERP-components like P1, P3, and LPC (late positive component). The LPC is a positively going ERP-slow wave which has been found particularly sensitive to two types of stimulation. On the one hand, the presentation of emotional stimuli of negative versus positive valence (e.g. [30, 36, 51, 59]) revealed an enhancement in LPC-ativity. On the other hand, stimulus-conflict between neutral non-emotional stimuli, such as incongruent versus congruent color-word conjunctions in a Stroop task, also revealed an increased LPC for the conflicting condition (e.g., [13, 18]). Comparing these components among our four cueing conditions allowed us to estimate both the effects of emotional content presented in a single modality and redundant “emotional cueing” in both modalities (Fig. 1).

## Results

### Behavioral results

Our main research interest was to investigate whether audio-visual cues with bimodally fearful coloring would enhance attentional shifts toward their location compared to audio-visual cues with only a single modality of fear-related display or a fully bimodal neutral display. Participants were instructed to ignore any bimodal face-voice cue and to attend only to the arrow targets. Due to problems with the presentation of the auditory cues in the first participant, this first data set was excluded from the further analyses.

**Fig. 2:**
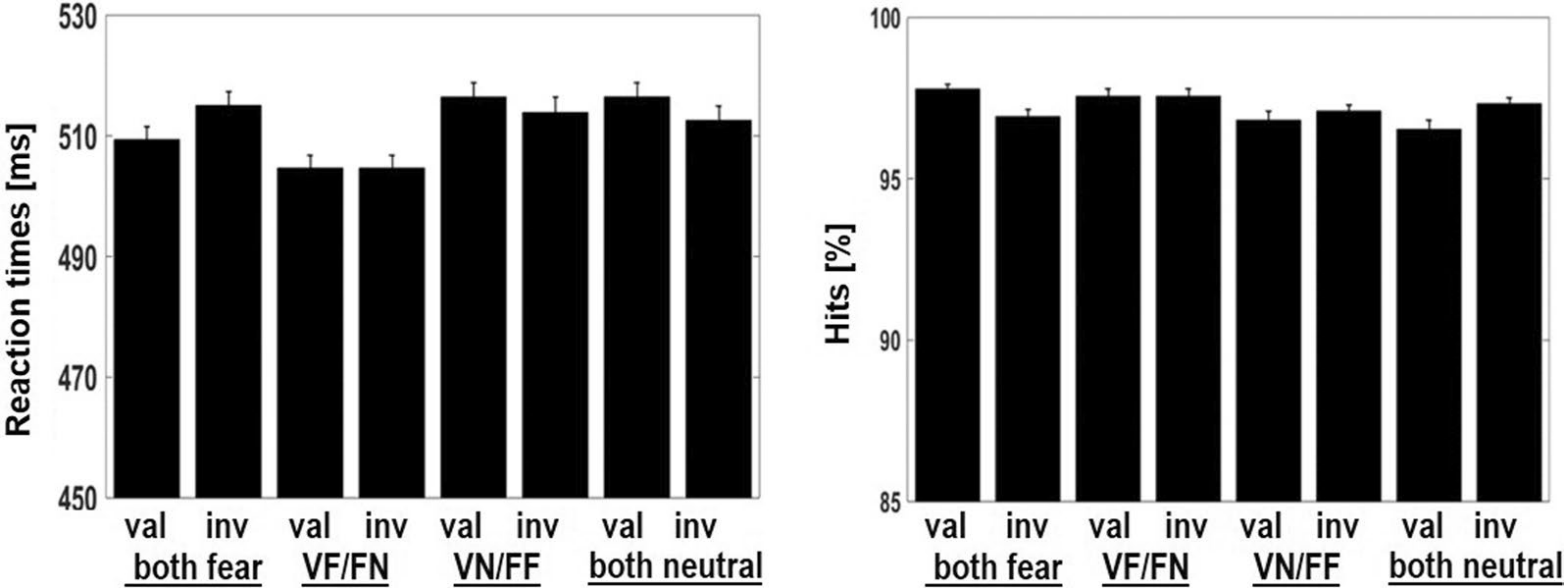
Behavioral results. Left panel: Reaction times. The interaction of cue-type with validity was significant, mainly due a significant validity effect with valid faster than invalid combinations in the both fearful condition, but there was a trend for a reversed validity effect in the neutral condition. The validity difference of the both fear condition was significantly larger than the inverted one in the both neutral condition. Right panel: Hit-rates: Despite not being significant, the hit-rates mirrored a similar interaction, with improved performance after both fearful cues for valid targets, but for invalid after neutral targets. *both fear *face/voice both fearful, *VF/FN* voice fearful with neutral face, *VN/FF* voice neutral with fearful face, *both neutral* face/voice both neutral)

The analysis of reaction times of trials associated with a correct response failed to yield a significant main effect of VALIDITY (F(1, 24) = 0.013; p = 0.909; ηp^2^ = 0.00063). The interaction of EMOTIONAL CUE COMBINATION and VALIDITY was significant (F(2.525, 72) = 3.363; p = 0.024; ηp^2^ = 0.138). The follow-up Bonferroni-corrected posthoc tests indicated that the participants responded significantly faster in the valid than the invalid condition when both face and voice were fear-related (t(24) = 3.208; p_bonf = 0.034), but there was no significant validity effect following any other emotional cue combination. In addition, the main effect of EMOTIONAL CUE COMBINATION was significant (F(2.083, 72) = 4.268; p = 0.019; ηp^2^ = 0.169). Bonferroni-corrected post-hoc tests demonstrated first that reaction times after the voice-fear/face-neutral cues were faster than after voice-neutral/face-fear cues (t(24) = 3.182; p_bonf = 0.014). Secondly, reaction times to targets after the voice-fear/face-neutral cues were also faster compared to targets after the both neutral face-voice combinations (t(24) = 2.988; p_bonf = 0.024) (Fig. 2).

Considering hit-rates, neither the interaction of the factors nor any single main effects were significant.

### ERP results

The main aim of the study was to test whether emotion shifts spatial attention more strongly when presented in two instead of one modality. To test, bimodal face-voice cues consisted either both of fearful expression, or only one of a fearful expression, or both with a neutral expression (control condition) (Fig. 2).

#### Contra- versus ipsilateral target activity at P1

**Fig. 3:**
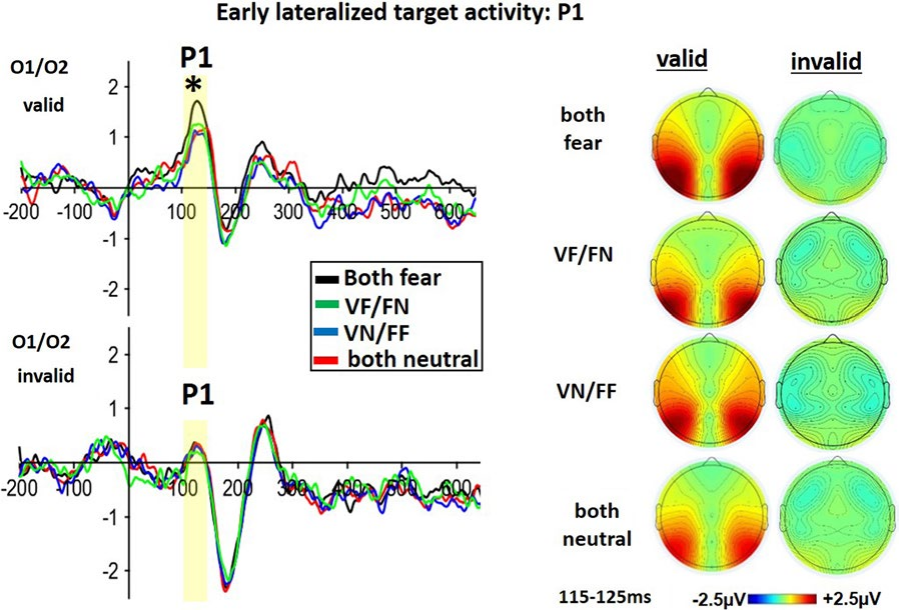
Early lateralized target activity: P1: Left panel: ERP-traces time-locked to the target over occipital O1/2-electrodes, the contra-versus-ipsi-lateral P1 amplitudes indicated a significant increase for all valid compared all invalid targets (see upper versus lower part of the figure). In addition, for valid conditions (upper channel), the contra-ipsilateral P1 activity of targets after the double fear combination was significantly increased compared to targets after combinations of both neutral or of neutral-voice with fearful face combination (VN/FF). The comparison of the valid double fear combination with the valid fear-voice/neutral-face combination (VF/FN) only showed an increase by trend, but was not significant. Right Panel: Topographies indicate the corresponding contra-versus-ipsi-lateral P1-differences separately for in/valid targets and its associated cue-type. Contra-versus-ipsi-lateral amplitude differences are summarized over hemispheres

**Fig. 4:**
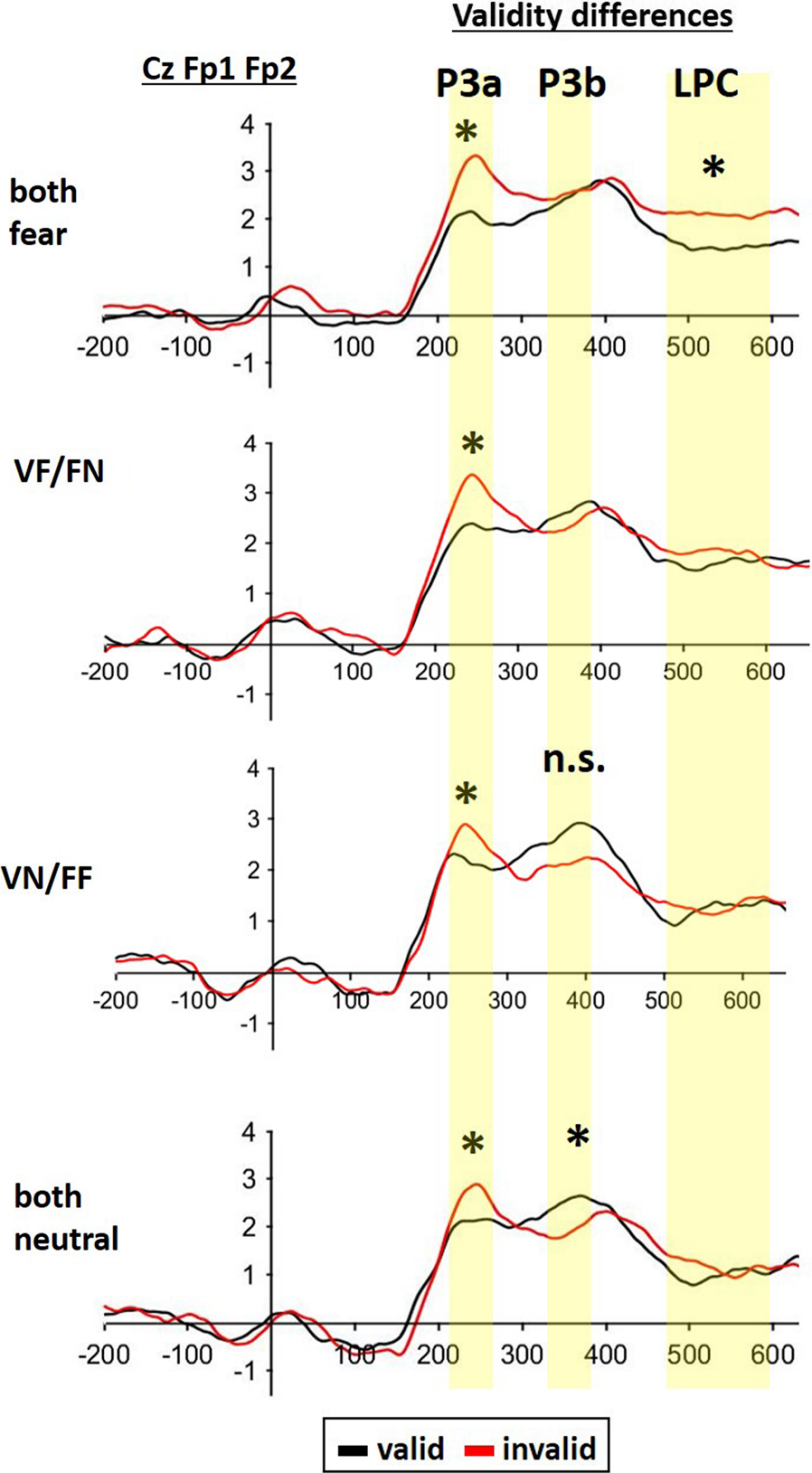
ERP-traces of late centralized target activity time-locked to the target over the central-parietal ROI. P3a: A significant main effect of validity was revealed, indicating that P3a-activity was increased for the invalid versus valid condition independent of cueing type. P3b: The interaction of validity by cueing type was significant. Posthoc-tests indicated that validity differences were only significant for the double neutral (VN/FN) condition with a reverse effect of higher P3b amplitudes for the valid instead of invalid condition. LPC: Again, the interaction of validity by cueing type was significant. Posthoc-tests indicated that validity differences were only significant for the double fear (VF/FF) condition an increased LPC-amplitude for the invalid versus valid condition

We analyzed the P1 amplitude as contra-ipsilateral activity differences over occipital electrode positions. First of all, the 4 × 2–ANOVA EMOTIONAL CUE COMBINATION (both fear, voice fear/face neutral, voice neutral/face fear, both neutral) and VALIDITY (invalid, valid) was calculated (see Fig. 3). It revealed a significant main effect of VALIDITY (F(1,24) = 27.844; p < 0.001; ηp^2^ = 0.537) with larger P1-activity for valid compared to invalid cue combinations. The main effect of EMOTIONAL CUE COMBINATION was not significant (F(2.928, 70.277) = 2.088; p = 0.109; ηp^2^ = 0.080). Importantly, the interaction of both factors was significant (F(3, 72) = 2.888; p = 0.038; ηp^2^ = 0.169). Due to further investigation of the significant interaction, two follow-up ANOVA’s considered valid and invalid P1-activity respectively. Only the ANOVA for the valid conditions indicated a significant effect of EMOTIONAL CUE COMBINATION (F = (3,72) = 3.347; p = 0.040; ηp^2^ = 0.176; see Fig. 4). A Bonferroni-corrected posthoc test revealed that the contra-ipsilateral activity of validly cued targets after the both fear combination was significantly increased compared to targets after the both neutral face-voice combination (t(24) = 3.012; p_bonf = 0.020). A further Bonferroni-corrected posthoc-test showed in addition a significant increase of ipsilateral activity of validly cued targets when comparing the both fear combination to the neutral-voice with fearful face combination (VN/FF; t(24) = 3.206; p_bonf = 0.019). The corresponding posthoc comparison of targets after the both fear combination versus targets after the fear-voice with neutral-face (VF/FN) combination was not significant, despite of showing a trend (t(24) = 1.425; p_bonf = 0.083).

#### Centralized target activity at P3a and P3b

**Fig. 5:**
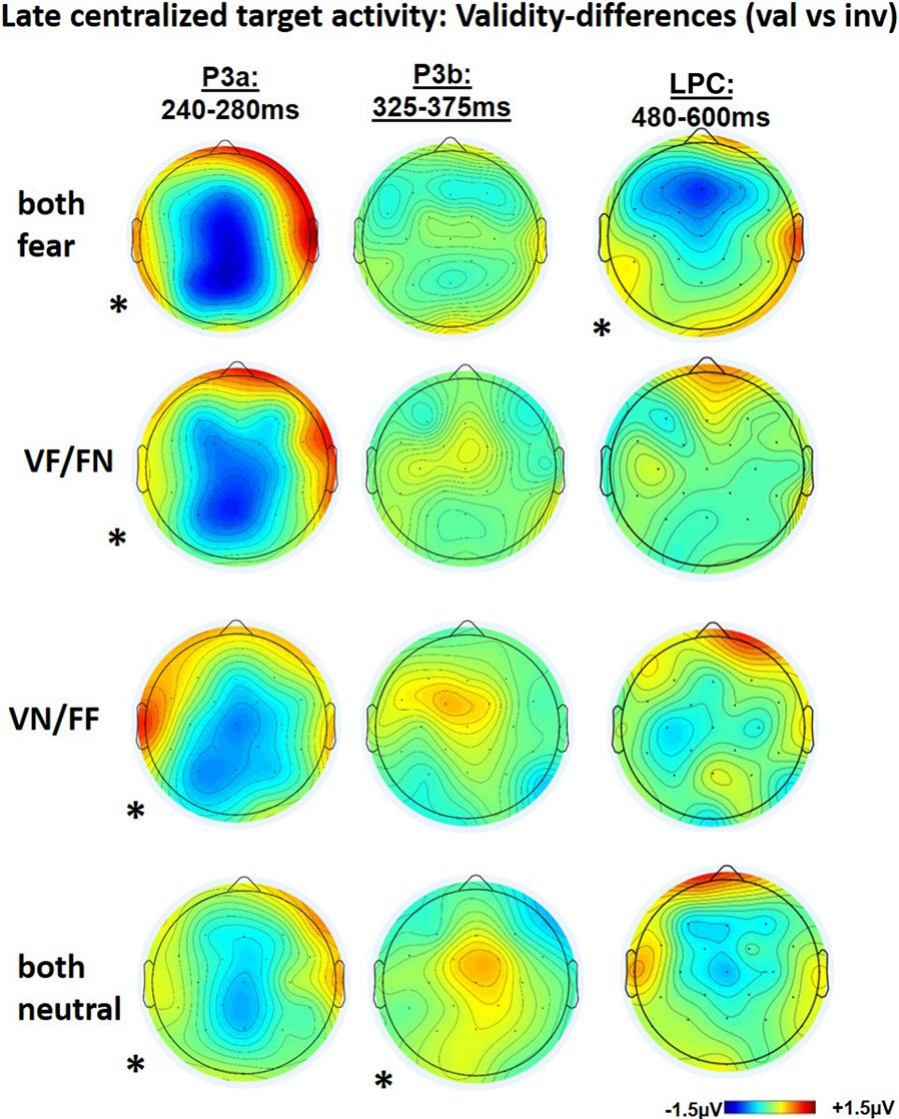
ERP-topographies of the late centralized target activity showing validity differences of valid minus invalid conditions time-locked to the target over the central-parietal ROI. For P3a, a significant main effect of validity differences indicated an increased P3a-activity for the invalid condition independent of cueing type (see blue activity color). For P3b, validity effects were only significant for the double neutral condition (VN/FN) and indicated a reverse validity difference with increased valid activity (see orange activity color). For LPC, validity differences were only present in the double fear condition (VF/FF) increasing for the invalid versus valid condition

We analyzed the modulation of P3a amplitude at the central-parietal ROI (Cz/Fp1/Fp2). Neither the interaction of EMOTIONAL CUE COMBINATION and VALIDITY (F(2.391,72) = 1.970; p = 0.126; ηp^2^ = 0.076) nor the main effect of EMOTIONAL CUE COMBINATION (F(1.833, 72) = 2.043; p = 0.145; ηp^2^ = 0.078) were significant. However, there was a significant main effect of VALIDITY (F(1,24) = 13.375; p < 0.001; ηp^2^ = 0.358; Figs. 4, 5).

Amplitude values at the P3b revealed a significant interaction of EMOTIONAL CUE COMBINATION by VALIDITY (F(2.27, 65.05) = 2.67; p = 0.049; ηp^2^ = 3.262). Follow up Bonferroni-corrected posthoc tests indicated that validity effects were only significant in the neutral condition (t(24) = 3.088; p_bonf = 0.045), but in none of the other face-voice combinations (Figs. 4, 5). None of the other effects reached statistical significance.

#### Centralized target activity at LPC

Additionally, we analyzed the modulation of LPC amplitude at the central- parietal ROI (Cz/Fp1/Fp2). The interaction of EMOTIONAL CUE COMBINATION and VALIDITY was not significant (F(3, 72) = 1.103; p = 0.354; ηp^2^ = 0.048). However, there was a significant main effect of EMOTIONAL CUE COMBINATION (F(2.155, 47.421) = 4.743; p = 0.012; ηp^2^ = 0.177) with Bonferroni-corrected post-hoc tests indicating that the both fear combination revealed significantly higher LPC activation than the both neutral combination (t(24) = 3.226; p_bonf = 0.023), whereas all other combinations were not significant. Further, there was a significant main effect of VALIDITY (F(1, 24) = 4.233; p = 0.049; ηp^2^ = 0.161). Importantly, Bonferroni-corrected posthoc-tests indicated only a significant validity difference in the bimodal both fearful condition (t(24) = 0.024; p_bonf = 0.024), whereas all other combinations failed to reach significance.

#### Cue-related N170 activation

**Fig. 6:**
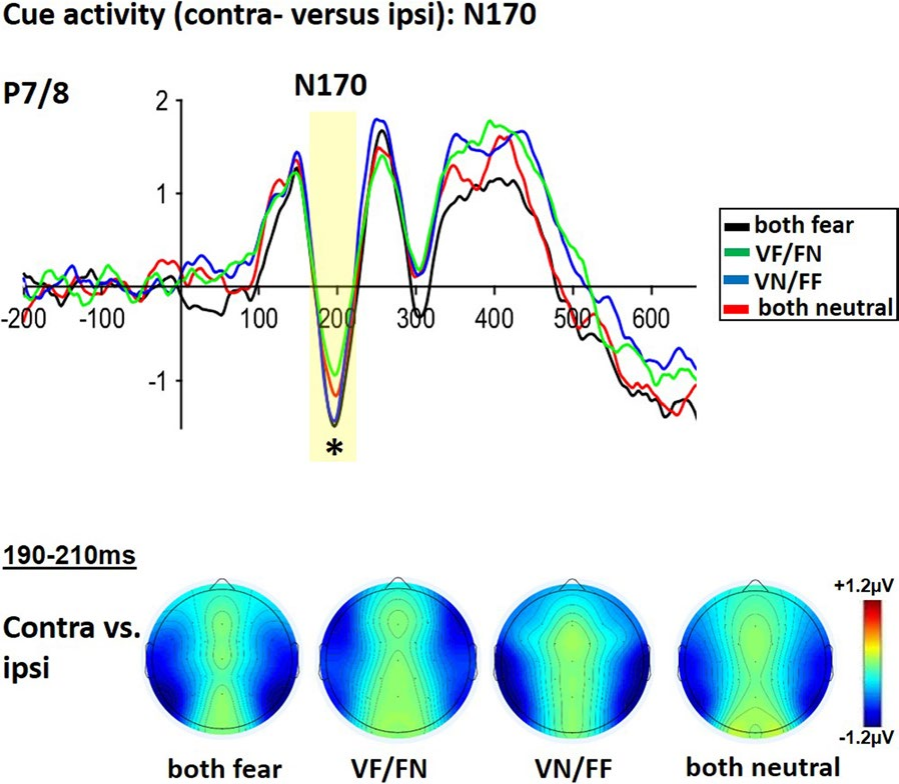
Cue activity (contra- versus ipsi): N170: upper panel: ERP-traces time-locked to the cue, the contra-versus ipsi-lateral N170 amplitudes were increased when face-voice combinations included a fearful face. Lower panel: Topographies over each hemisphere summarize the contra-versus-ipsi-lateral activation corresponding to N170 at 190-210 ms

Specifically asking for spatial effects due to the varying emotional presence during the cue-target interval, we analyzed contra-versus-ipsilateral N170 activity differences over occipital-parietal electrode positions (P7/P8) separately for each emotional cue combination. ANOVA results showed a significant main effect of emotional cue combination (F(3, 72) = 2.807; p = 0.046; ηp^2^ = 0.105 see Fig. 6). Bonferroni-corrected posthoc-tests indicated that the N170 for the both-fear combination was significantly more negative than for the voice-fear with face-neutral combination (VF/FN; t(24) = 3.233; p_bonf = 0.012; see Fig. 6). Similarly, also the face-fear with voice-neutral combination (VN/FF) revealed an increased N170 compared to the voice-fear with face-neutral combination (VF/FN; t(24) = 2.939; p_bonf = 0.045). Finally, the both-neutral combination was not significantly different when compared to any other combination.

## Discussion

The current ERP-study investigated whether double emotional face-voice cues attract attention to their location more strongly than single emotional cues or purely neutral cues, respectively. To test, bimodal face-voice cues that varied concerning their degree of emotional content; i.e., fearful in face and voice (both fearful), fearful voice/neutral face or fearful face/neutral voice (single emotional cue), or both neutral (both neutral cue) preceded a visual target. Considering reaction times, the number of cue components related to fear (both, one, none) interacted with cue validity. This was mainly due to a pronounced validity effect in the both fearful condition. Target related ERP-effects started with a contra-versus-ipsi- lateralized P1 increase for valid versus invalid conditions which was largest for the both fearful condition. The early P3a showed a main effect of increased activity in invalidly cued compared to validly cued trials, independently of presence and quantity of emotional content. In contrast, the P3b component was, again, differentially affected by the type of cue, displaying significantly larger amplitudes after valid than after invalid cues (i.e., reversing the P3a pattern) for all cueing conditions except for the bimodal fearful condition. The former pattern of results (i.e., P3a larger after valid than after invalid cueing, P3b larger after invalid than after valid cueing) thus resembles the course of reaction times over the cue-target interval, that is, a regular validity effect after short intervals which turns into a reversed validity effect after longer periods. Viewed from this perspective, the absence of the reversal effect in the P3b for the both fearful condition would be consistent with the assumption of a reduced IOR mechanism. Finally, yet importantly, only in the both fearful condition, there was an increase of LPC for invalid compared to the valid condition, possibly indicating that evaluation of the emotional content is enhanced by a mismatch of spatial positions.

### Performance data

The present behavioral results indicated that emotional content interacted with spatial validity. Regarding reaction times, this interaction was mainly driven by a strong validity effect in the both fearful condition, contrasting with a descriptively slightly reversed validity effect of the neutral condition. Although the hit rate analysis did not yield any statistically significant interactions (which might not be surprising given the overall high level of accuracy), the result displayed a trend of improved performance after double fear cues for valid targets, and for invalid targets after neutral cues. Therefore, the interaction trend in the hit rates resembled the result pattern observed in reaction times. Enhancement of the cueing validity effect in the both fearful condition corresponds to previous findings using unimodal fearful cues. As previously shown, fearful facial expressions [72] as well as fear-related voice cues [74] enhanced performance differences in responding to targets presented at validly versus invalidly cued positions. Importantly, this cueing validity effect was found not only with short SOA for face cues (117 ms) but also with long SOA for voice cues (1200 ms), as long as the ISI was kept short, suggesting that the occurrence of IOR depends on a long ISI. The results of the current study seem in line with this assumption as there was no significant reversal of the validity effect—and a significant regular validity effect in the both fearful condition—despite the SOA exceeded 1000 ms.

Further, effects of bimodal cues have so far only been tested lacking emotional content [47, 63]. In the study by Santangelo et al. [63], bimodal cues evoked a statistically non-significant behavioral enhancement of target processing compared to unisensory cues. In the study of Mastrobernadino et al. [47], enhanced validity effects were observed for bimodal cueing (i.e., correspondence of the picture of a laterally presented animal face occurring alongside another animal face on the opposite side with a centrally presented sound) but these effects were confined to conditions of high task difficulty. While this may be the case for non-emotional cues, the results of our study show that this is not the case for emotional material. Our task consisted of an easy discrimination of a lateralized arrow pointing up- or downwards. According to the overall high accuracy rates of our participants (98.3%), this is a task of rather low perceptual demands. Instead, the results of the current study suggest that bimodally presented fear-related content of spatial cues may be sufficient to enhance the deployment of spatial attention to the cued position compared to bimodal non-emotional cues.

Cues of both neutral content indicated a slight non-significant tendency for an opposite cueing effect of improved performance for invalid compared to valid targets. In the current study, the sound duration was set to 1000 ms to ensure the discrimination of the auditory content. Together with the current ISI of 190–240 ms, the SOA-time was therefore 1190–1240 ms. Considering neutral sound cues of similar psychophysical structure [75], we previously found typical spatial validity effects (i.e., improved performance to valid compared to invalid targets) with an interstimulus interval (ISI) of 50–150 ms, but SOA of 1050–1150 ms due to the long sound duration. Importantly however, neutral cueing effects inverted in an IOR-like manner (invalid improved compared to valid) with an ISI of 650–750 ms, while an ISI of 350–450 ms indicated no cueing effects in neither direction [75]. In the current study, the ISI was 190–240 ms, thus outside the time-range of typical validity effects for neutral cues as found in our previous study. It would thus seem straightforward to assume that the interval administered in the current study was too short to yield reliable IOR, while, on the other hand, sufficiently long for substantial undoing of the initial attention shift towards the cued location, except for the bimodal fearful cueing condition, in which disengagement of attention from the cued location was less feasible.

#### Validity differences for targets: ERP-results

##### Lateralized contra-vs-ipsi P1-effect

Time-locked to targets, contra-versus-ispi-lateralized P1-activity indicated an enhancement of valid versus invalid activity for all conditions. Importantly however, targets after bimodal fearful cues indicated a specifically large P1-validity difference due to a massive activity increase for validly cued targets. One the one hand, the general input of P1 for all valid vs. invalid conditions fits well with a spatial bottom-up theory where early target processing is enhanced at positions preoccupied validly by spatial cues independent of awareness or SOA duration [27, 54, 72]. For example, in a spatial cueing paradigm, Giattino and colleagues [27] used lateralized cues of short 17 ms duration hidden in a multiobject-presentation. Targets consisted of neutral quadratic shapes at valid or invalid positions of the cues. Independently of whether participants consciously perceived the cues or not, validly cued targets always evoked higher P1-activity over occipital areas than invalidly cued targets. In addition, in a spatial cueing study with facial anger cues and neutral targets, Liu et al. [44] found that target-related P1-activity increased when validly versus invalidly cued. Similarly, Brosch and colleagues [8] found increased P1 for valid vs. invalid targets lateralized to side of the fearful facial expression when double facial cues with one fearful and one neutral expression were presented. In addition, P1-valdity differences were also found in crossmodal spatial cueing when an auditory anger voice cued a visual neutral target [7]. All these P1-validity differences occurred early (around 100 ms) and showed a strong responsiveness of the spatial location of the target in relation to the preceding position of the emotional cue [8, 72]. These effects might therefore mirror a bottom-up process of sensory processing [8, 72]. Our current finding supports this hypothesis by extending previous data with a specifically contra-vs-ipsilateral P1-validity difference and its enhancement by bimodally double fearful stimulation.

##### Central P3a-effect

ERP-data time-locked to targets, P3a-activity increased for invalid versus valid double combinations independent of condition. Thus, independent of emotional presence or absence, spatial validity corresponded inversely to the P3a-activity. With spatial cueing the P3a has been so far mainly investigated with predictive designs (i.e., 75% valid vs. 25% invalid conditions), where the low probability of invalid stimuli increases their novelty character of spatial position [16, 24, 28, 29]. However, while the P3a validity differential activation might be larger in predictive cueing, it was still present in the current unpredictive cueing design (50% valid/invalid). The reason might be that even in unpredictive cuing an invalid target position still needs to be spatially updated compared to the preceding cue position, thus leading to increased P3a activation for invalid versus valid targets. Similarly, Lasaponara and colleagues [40] found increased invalid P3a activity when using only a 50% ratio of valid stimulation compared to the sum of invalid and no-cue conditions. Considering the modulation by emotional content in spatial cueing, there is often mentioned only a general P3-activity without subdivisions of P3a and P3b [21, 44, 73, 75]. These emotional ERP-studies mainly revealed P3 validity differences that increased in positive activity for invalid versus valid target stimuli when cued with fearful faces [21, 44]. Importantly, when targets were preceded by disgust sounds, such P3 effects inverted independently of the ISI-length, indicating an increased valid versus invalid activity, which corresponded to early shifts of attention away from the disgust evoking stimulus and may therefore relate to an automatic early bottom-up process of emotional context [73, 75]. As fearful like neutral stimulation supports processing of targets at the valid versus invalid position, the early spatial P3a-effects may also point to automatic early bottom-up processes.

##### Central P3b-late effect

From 325 to 375 ms, the both neutral condition and the fearful face with neutral voice condition (VN/FF) revealed validity effects with increased valid versus invalid activity. Firstly, considering the above-mentioned P3a, these P3b validity-effects were inverted in polarity as they now indicated more activation for valid than invalid targets. This might indicate a disturbance by the valid instead of invalid spatial position and fits well with the current behavioral data indicating an IOR-tendency for the fully neutral condition and VN/FF condition. Secondly, IOR-effect with neutral stimuli can be found over occipital-parietal electrodes at P3-time intervals [48, 58, 75], indicating that attention needed to be boosted at the validly instead of shifting away to the invalidly cued and IOR-conform position. Thirdly, some recent research [39, 62] suggested that a late P3-activity mirrors conscious perception. Thus, the relatively long delay of target-onset after the neutral and mixed VN/FF-cues might support an expectancy effect of target processing at the invalid position. In conclusion, the validity inversion of the late P3, respectively P3b, seems to fit well with the behavioral occurrence of a probable IOR-effect that is missing when full emotional bimodal integration is required.

##### LPC-activity

At around 500 ms time-locked to targets, a positive slow wave (late positive component; LPC) showed validity differences exclusively in the bimodal both fearful combination again with an enhanced invalid versus valid activity. This finding seems to combine different views on the LPC (late positive component); a component that is sometimes thought to include the late positive potential (LPP; [12] for a review). Firstly, the LPC might be considered as a slow wave conflict potential [13, 18, 50]. For example, the LPC-activation appears in non-emotional Stroop-tasks as well as non-emotional Flanker-tasks and mirrors the conflict in semantic meaning (Stroop) of an incongruent color-word-combination relative to congruent color-word-combination respectively the perceptive conflict (Flanker) of incongruent versus congruent target-flanker combinations [1, 13, 18, 42, 70]. Secondly, the LPC/LPP is handled as an indicator of negative emotional processing [30, 36, 51, 59]. In this sense, the LPC/LPP increased over centro-parietal scalp regions for negative versus neutral emotional stimuli, when ERP-studies required a simple emotional discrimination of a centrally presented picture [30, 36, 51, 59]. In addition, a central cueing study [33] indicated validity effects in the same direction as the current study, e.g., increased LPP-activity after invalidly compared to validly cued negative emotional pictures. In the aforementioned studies, the conflicting or negative enhanced LPC was time-locked to the onset of the emotional stimulus itself. In contrast, the LPC difference in the present data was found time-locked to the presentation neutral target, importantly however when following double negative cues and indicating spatial conflict. In addition, perception of stimulus conflict might yield aversive states ([17], for a review), and fits therefore well to the just cued fearful bimodal perception. More specifically, the LPP/LPC complex is thought to be influenced by top-down processes (e.g., reinterpretation of negative stimuli) and reflecting endogenous spatial attention (cf. [12], for a review). Thus, our data might integrate and extend present LPC-theories by showing that targets, invalidly cued by fearful bimodal stimuli, are associated with enhanced spatial conflict.

##### Cue-related N170-activity

Time-locked to cues, the facial related N170 component was always enhanced when the facial cue expressed fear—independent of the accompanying sound expression. Investigating bimodal effects of neutral face-voice combinations, Latinus and colleagues [41] found that the N170 did not differ between purely facial and combined facial-voice stimuli. Considering emotional influence, purely visual spatial cueing studies found that N170 activity increased for fearful versus neutral facial spatial cues independently of whether their emotional content was task-relevant [11, 69] or task-irrelevant ([15]; see also [31], for a review). In contrast, some facial studies (e.g., [8, 55]) used a double facial cue with one emotional and one neutral face. In this case, emotional enhancements at N170 might be rather hard to detect or at least weakened by necessary subtraction analysis to extract fear-related activation. In addition, asking for overt discrimination of facial emotion or gender, emotional discrimination also revealed an enhanced N170 for fearful compared to neutral expressions [3, 4, 61], but see [19] for no N170 differences). So far, bimodal emotional differences of N170 have only been investigated with matching emotional face-voice combinations (e.g., bimodal fearful or bimodal neutral), revealing that N170 was enhanced for bimodally fearful compared to bimodally neutral face-voice combinations [68]. Only an increase in the N170 amplitude for angry versus neutral faces, however independently of voice, was found. Therefore, the present finding extends the results of previous studies, by emphasizing the importance of facial expressions for the N170, and its independence of the accompanying voice content. A possible explanation might relate to the visual discrimination demands of the experimental setting, where movement of the attentional focus is dominated by visual information and an added voice might unspecifically enhance attention in general, at least at this early time of processing.

### Limitations

Contrasting the spatial effects of fear and neutral content of bimodal cues, this study consisted of a 4 × 2 stimulus paradigm and thus eight conditions (e.g., even 16 conditions when splitting into ipsi- and contralateral activity). To ensure a relatively high signal-to-noise ratio of the ERP-data (see [6]), we decided to confine experimental manipulations to a single ISI and a single type of emotional content. This resulted in two noteworthy caveats. First, adding a second even longer ISI might have yielded stronger evidence for our interpretation of a delay of disengagement of attention from the cued location in the both fearful condition (i.e., if consistent IOR for all conditions could be observed with a sufficiently long SOA). Second, it should be stressed that our experiment was confined to the investigation of one particular emotion (i.e., fear) and that the results may not transfer to visual-auditory cues related to other emotions. As noted in the Introduction, studies of spatial cueing featuring disgust-related cues yielded a reversed validity effect (i.e., superior performance in invalidly cued trials), consistent with a repelling or dispersing effect on spatial attention (e.g., [44, 72, 74]). Assuming that the validity effect in the bimodal fearful condition of the current study reflects a reduction in attentional disengagement counteracting IOR does not seem to allow predictions for situations in which attentional engagement is absent in the first place.

## Conclusions

The present behavioral and ERP-data compared for the first time spatial cueing effects elicited by bimodal face-voice cues of varying emotional content (both fearful, face fearful with voice neutral, face neutral with fearful voice, both neutral). Typical spatial cueing effects with faster reaction times to validly cued targets than to invalidly cued targets selectively occurred in the both-fearful condition. This behavioral effect corresponded to P1-validity differences of target processing, that were largest in the both-fearful condition, as well as to a later LPC-validity difference, which was exclusively present in trials with bimodal fearful cues. In contrast, an early general main effect of validity in the P3a component indicated that spatial attention was initially directed towards the validly cued spatial position even in trials involving both neutral or single emotional types (albeit less pronounced than in the both fearful condition). The pattern of the P3b component suggested that spatial attention was re-directed, but to a lesser extent in the bimodal both-fearful condition. In conclusion, our present data therefore suggest that, at least when using long SOAs with short ISI, only double fearful stimulation prevented or at least delayed the disengagement of spatial attention from an invalidly cued position. Single emotional stimulation, by contrast, did not counteract such disengagement and presumably the initiation of IOR, compared to completely neutral stimulation, in a noticeable manner. Future bimodal cueing studies might corroborate this conclusion by extending the length of the ISI. Selective reduction of attentional disengagement from a cued location after bimodal emotional cues should evidence itself in selective absence or delay of IOR in this condition (see [23], for a similar approach concerning the impact of emotional cue content in general).

## Methods

### Participants

Planning of sample size was based on previous, comparable spatial cueing studies that investigated the modulatory effects of emotion [73, 75]. As behavioral effects were not evident in all studies [63], P1 differences between valid and invalid trials for both fearful face-voice cues were considered the relevant endpoint. Indeed, a post-hoc analysis with G*Power [20] showed that the achieved power in our study was 1-β = 0.99 given the found difference between P1 peaks (two-tailed t-test, α = 0.05, dz = 1.04) Provided the found effect size in this study (dz = 1.04), analysis with G*Power indicated that a sample size of 10 participant is sufficient to detect a significant difference with 1-β = 0.80, α = 0.05.

Twenty-six participants (12 men, *M*_age_ = 23.4 years, SD = 4.5) took part in the ERP-experiment. All were right-handed, had normal or corrected-to-normal vision, normal hearing abilities and had no history of psychiatric or neurological disease. One of these participants was excluded from the final data analysis due to poor quality of the EEG signal (excessive movement and drifts). All participants were students of the MSH and received study-participant-points. They gave written informed consent according to the ethical standards laid down in the Declaration of Helsinki (BMJ 1991; 302; 1194). The study was approved by the Ethics committee of the MSH.

### Materials and procedure

We used a spatial cueing paradigm with bimodal face-voice cues to investigate if and how congruent emotional content in two modalities would lead to stronger engagement to the cue and therefore to enhanced target processing when compared to single emotional presence in only one modality or purely neutral combinations. Importantly, face-voice combinations were always bimodal cues, consisting of a facial expression with a non-verbal voice expression. Congruent combinations consisted of either two fearful expressions or two neutral expressions, whereas single emotion combinations included one modality fearful and the other neutral. During the entire EEG-experiment, a fixation cross was presented at the center of the screen to ensure exogenous and peripheral stimulation. Each trial started with the presentation of a bimodal face-voice cue for 1000 ms. On the offset of the face-voice cue followed a jittered cue-to-target-interval of 190-240 ms. Then a visual neutral target (a little white arrow pointing up or down) was presented for 100 ms. After an intertrial interval of 2–3 s, the next trial started (see Fig. 1). The task of the participants was to ignore the bimodal face-voice cues and to respond to the pointing direction (up/down) of the target. All participants completed 10 runs. Each run consisted of 60 trials and lasted about 3.5 min, leading to a total experimental run time of about 35 min.

#### Stimuli

The emotional cueing voice was either a fearful voice (someone fearfully screaming) or a neutral voice (someone biting into an apple). These voices were already used and rated in our previous studies (e.g. [73, 74]). In our previous study [74], the fear voice was rated as clearly negative (valence) with high arousal, whereas the neutral voice was rated as neutral (valence) with low arousal. Each of the two voice stimuli had a duration of 1000 ms. This duration guaranteed that the emotional content of the voice was fully processed by the participants before the presentation of the target (e.g., [52]) and was also chosen in our previous studies [73, 74]. The overall sound level was normalized to 68 dB SPL for both emotional voices. To preserve the emotional character of the voices, the time–frequency structures of both voices were not changed (cf. for a similar procedure with happy/sad emotional voices: see [2, 34, 35]). For lateralized presentation, the originally stereo-recorded voices (someone fearfully screaming, someone biting into an apple) were converted into mono-channel sounds by using “Au Adobe Audition” (http://www.adobe.com). During ERP/EEG, these mono-channel sounds were then delivered using the software Presentation (neurobehavioral systems; http://www.neurobs.com) to either the left or the right loudspeaker. Both loudspeakers were positioned behind the right and left side of the screen to induce an association between voice and target location. We did not use headphones due to their interference with the EEG-cap and -measurements. Similarly, the emotional faces consisted either of a fearful or neutral expression. After the EEG-measurements, participants described the facial expressions in their own words and applied ratings of emotional valence and arousal. First, they were asked to name the facial expression (open answer). Indeed, the fearful expression was described with fear-related words (e.g., “fearful”, “frightenend”, “horrified”, “panicking”) by 88% of the participants (22 out of 25) as well as the neutral expression with non-emotional related words (e.g., “neutral”, “bored”, “well-balanced”) by 88% of the participants. Secondly, participants rated valence and arousal of the facial expressions on a scale ranging from 1 to 5 (from lowest arousal/negative valence to highest arousal/positive valence). For statistical analyses, the ratings for valence and arousal, respectively, were averaged across participants for each facial expression. Paried t-tests indicated more negative valence (t(24) = 14.000; p < 0.001) and higher arousal (t(24) = 6.385; p < 0.001) for the fearful than for the neutral expressions. To ensure fully bimodal stimulation during the EEG-experiement, onset and offset of sounds and faces was simultaneously.

#### EEG recording

The BrainVision Recorder (Brain Products, Germany http://www.brainproducts.com/) was used for recording EEG/ERP-data in combination with a 32 channels electrode cap of the electric shielded EEG-system “actiCAP snap”, (Brain Products, Germany). According to the 10–20 EEG system, the 32-electrodes included seven frontal electrodes (FP1, FP2, F7, F3, FZ, F4, F8), four fronto-central electrodes (FC5, FC1, FC2, FC6), nine central-temporal electrodes (T7, C3, Cz, C4, T8, CP5, CP1, CP2, CP6), four lateral electrodes (FT9, FT10, TP9, TP10) and eight parietal-occipital area electrodes (P7, P3, PZ, P4, P8, O1, OZ, O2). All 32 electrodes were referenced to the algebraic average of all channels and were therefore unbiased to any electrode position. The ground electrode was placed between electrode position Fp1 and Fp2. Electrode impedances were kept below 10 kΩ for all electrodes. A dimly lit, sound-attenuated and electrically shielded chamber was used for EEG-recording.

#### Behavioral data

Our main interest was to find evidence for enhancement of spatial cueing effects by emotional coloring in two versus one or none sensory modalities of a bimodal audio-visual cue. Only trials with behavioral responses between 200 and 1000 ms after the presentation of the arrow-target were considered for further behavioral analysis (98.3% trials in total). For all cue conditions, reaction times (RTs) to correctly judged arrow-targets as well as accucary rates were analyzed. Using the statistic program JASP version 0.16.1.0 (https://jasp-stats.org/), for each performance measure (RTs respectively accuracy rates), a repeated-measures 4 × 2 ANOVA was performed with the within-subject factors EMOTIONAL CUE COMBINATION (both fearful, voice-fearful with face neutral (VF_FN), voice-neutral with face-fear (VN_FF, both neutral) and VALIDITY (valid, invalid). Greenhouse–Geisser correction was applied to all statistical comparisons for which the Mauchly test indicated violation of sphericity. In case of significant interaction, follow-up posthoc Bonferroni corrected t-tests were used to indicate improvements versus deterioration between single conditions. Significance was inferred for corrected p-values < 0.05.

#### ERP-data analysis

The custom ERPSS software (Event-Related Potential Software System, UCSD, San Diego, CA, USA, cf. [45] was added-on to the open source EEGLAB software (an open source environment for electrophysiological signal processing, UCSD, San Diego, CA, USA, cf. [14] and served as software for all ERP-analyses. ERP-analyses started by transforming the raw-data of the 10 runs of each participant into ERPSS format and combining them into one large data-file for further analysis. To avoid biasing to any electrode position, the reference for each of the 32 electrodes was set to the algebraic average of all channels. For cues as well as targets, the continuous EEG data were divided into 800 ms epochs, time-locked to the relating onsets including a pre-stimulus baseline of 200 ms. To discard epochs contaminated by large eye movements, excessive muscle activity, drifts, or amplifier blocking, an artifact rejection was performed by rejecting any voltage amplitudes under − 100 µV and over 100 µV. Resulting artifact-free EEG epochs were averaged together, separately for the various trial types (i.e., for cues: right receptively left sided cues for both fear/voice-fear with face-neutral (VF_FN)/voice-neutral with face-fear (VN_FF)/both neutral; for targets: invalid, receptively valid trial types corresponding to the four emotional conditions time-locked to the visual target). As a final step, all ERP-averages were digitally low-pass filtered (IIR-Butterworth) with a running-average filter of 30 Hz.

The aim of our study was to investigate if and how bimodally presented double emotional stimulation (face and voice both fearful) enhances reactions compared to bimodal audio-visual stimulations with fear solely in one of two sensory modalities. Target-related analyses focused on the early spatial P1-component as well as the later P3-component as indicator of attentional shifts [73, 75]. Further, the LPC-component was analyzed as an emotional and conflict indicator [33, 65]. For early target activity, we focused on contra-ipsilateral P1-differences at O1/O2-electrodes similarly to McDonald and colleagues (2013). Latency time window of 110–130 ms served as the P1 for all subsequent analyses of emotional variations in contra-ipsi differences. Contra-ipsi analysis was performed by using the contra-ipsi function of erplab [45]. This function first averaged each condition’s contra activation of right-sided stimulation at the left electrode O1 with its contra activation of left-sided stimulation at the right O2. Similarly, the ipsi-averages for each condition were calculated. In a second step, distraction of contra-versus ipsi-averaged stimulations was performed (cf. [45]).

For the P3 analysis, spatial cueing literature as well as our own studies had indicated that spatial cuing effects on visual targets are usually found over central-parieto-occipital electrode sites (for emotional cues: [44, 57, 73, 75], for neutral cues: [46, 49]). Thus, electrode positions located symmetrically over the visual-parietal cortex areas (Cz/Fp1/Fp2) were combined to a parietal-occipital region of interest (ROI). To select the time windows for theP3, in each subject, the average of all target conditions (i.e., averaged over validity and emotion appearance) indicated two peaks: a P3a-peak at around 260 ms and a later P3b peak at around 350 ms. For further analyses, these peaks were chosen for P3a and P3b with plus/minus 20 ms time windows. Also for the LPC-analyses, the ROI of the visual-parietal cortex areas (Cz/Fp1/Fp2; cf. [33]) was used. The time-window for the LPC was set to 480–600 ms, thus covering temporal LPC of previous literature [33, 65]. For the statistical analysis, we used mean amplitude values computed over the length of these intervals.

Cue-related analysis focused specifically on the ERP-component N170 as related to facial processing. The N170 is generally located over visual-parietal areas including electrodes P7/8, with N170 around 170 ms (e.g., [11, 69]; see [31] for a review). To test for attentional facial effects due to lateralized cue presentation, N170 differences were investigated by using contra-ipsi-differences. Contra-ipsi analysis was again performed by using the contra-ipsi function of erplab [45], however, this time comparing N170 ipsi/contra-activity of the left electrode P7 with the corresponding ipsi/contra-activity of the right electrode P8.

For all statistical analyses of the target-related P1- /P3- as well as cue-related N170 components, we used mean amplitude values computed over the length of the corresponding time-window intervals. All statistical ERP-analyses were performed by using JASP version 0.16.1.0 (https://jasp-stats.org/). For target-activity, two main analyses were performed separately. For the spatial P1-compenent, an repeated measurement 4 × 2 ANOVA included contra-versus-ipsi-activation for the factor EMOTIONAL CUE TYPE (fear/voice-fear with face-neutral (VF_FN)/voice-neutral with face-fear (VN_FF)/both neutral) and the factor VALIDITY (valid/invalid). To test for differences in both P3-peaks and LPC activation, for each component a similar 4 × 2—ANOVA with the factors EMOTIONAL CUE TYPE (both fear, voice fear/face neutral, voice neutral/face fear, both neutral) and VALIDITY (invalid, valid) was calculated, this time however including ERP-activation averaged over left and right target stimulation. For the N170 cue-locked activity, we analyzed whether and how the N170-amplitude of contra versus ipsi-stimulations differed between the stimulations of varying fearful content (cue with: both fear/voice-fear with face-neutral (VF_FN)/voice-neutral with face-fear (VN_FF)/both neutral). To test for differences in contra-ipsi activity at the N170, a repeated measure ANOVA including the factor EMOTIONAL CUE TYPE with the 4 repeated measures of the cue type (both fear/voice-fear with face-neutral (VF_FN)/voice-neutral with face-fear (VN_FF)/both neutral) was performed. For all analyses, significance was set to p < 0.05. Further, in case of significant results in the interaction of an ANOVA, subsequent posthoc-tests were added to indicate the cause of significance. Greenhouse–Geisser corrections was applied to all statistical comparisons for which the Mauchly Test indicated violation of sphericity.

## Data Availability

The datasets used and analyzed during the current study are available from the corresponding author on reasonable request.
